# Systematic analysis of lysine crotonylation in human macrophages responding to MRSA infection

**DOI:** 10.3389/fcimb.2023.1126350

**Published:** 2023-02-08

**Authors:** Hao Zhang, Wei Ma, Haoru Liu, Wanqi Tang, Junjie Shu, Jianping Zhou, Hongsheng Zheng, Hongyan Xiao, Xue Yang, Daoyan Liu, Huaping Liang, Xia Yang

**Affiliations:** ^1^ Department of Wound Infection and Drug, State Key Laboratory of Trauma, Burn and Combined Injury, Daping Hospital, Army Medical University (Third Military Medical University), Chongqing, China; ^2^ Deparment of Critical Care Medicine, Daping Hospital, Army Medical University (Third Military Medical University), Chongqing, China; ^3^ College of Basic Medical Sciences, Panzihua University, Panzihua, China

**Keywords:** proteome, host cells, multi-drug-resistant bacteria, crotonylated modification, response

## Abstract

Methicillin-resistant *Staphylococcus aureus* (MRSA) is one of the most commonly encountered bacteria found in healthcare clinics and has been ranked a priority 2 pathogen. Research is urgently needed to develop new therapeutic approaches to combat the pathogen. Variations in the pattern of protein posttranslational modifications (PTMs) of host cells affect physiological and pathological events, as well as therapeutic effectiveness. However, the role of crotonylation in MRSA-infected THP1 cells remains unknown. In this study, we found that crotonylation profiles of THP1 cells were altered after MRSA infection. It was then confirmed that lysine crotonylation profiles of THP1 cells and bacteria were different; MRSA infection inhibited global lysine crotonylation (Kcro) modification but partially elevated Kcro of host proteins. We obtained a proteome-wide crotonylation profile of THP1 cells infected by MRSA further treated by vancomycin, leading to the identification of 899 proteins, 1384 sites of which were down-regulated, and 160 proteins with 193 sites up-regulated. The crotonylated down-regulated proteins were mainly located in cytoplasm and were enriched in spliceosome, RNA degradation, protein posttranslational modification, and metabolism. However, the crotonylated up-regulated proteins were mainly located in nucleus and significantly involved in nuclear body, chromosome, ribonucleoprotein complex, and RNA processing. The domains of these proteins were significantly enriched on RNA recognition motif, and linker histone H1 and H5 families. Some proteins related to protecting against bacterial infection were also found to be targets of crotonylation. The present findings point to a comprehensive understanding of the biological functions of lysine crotonylation in human macrophages, thereby providing a certain research basis for the mechanism and targeted therapy on the immune response of host cells against MRSA infection.

## Introduction

The lack of effective drugs to eliminate multi-drug-resistant (MDR) bacterial infections and increasing number of infections are a key threat to public health worldwide (Berti and Hirsch, 2020). The World Health Organization (WHO) announced that annually, up to 10 million patients are expected to die from infections with MDR pathogens in 2050 (Merker et al., 2020). Methicillin-resistant *staphylococcus aureus* (MRSA), the most common Gram-positve pathogen in humans, has been reported to be responsible for more than 53 million infections worldwide (Peacock and Paterson, 2015) and more than 64% of patients infected with MRSA are likely to die (data from WHO). Multi-drug-resistant infection brings heavy burdens both economically and socially. Antibiotics show their therapeutic effects by disturbing major process of the bacteria, including cell wall synthesis, translation, transcription, and DNA synthesis (Vestergaard et al., 2019). Despite more than 60 years of clinically successful use of vancomycin, data suggest that vancomycin treatment is associated with adverse events, such as death and higher risk of nephrotoxicity (Rybak et al., 2020). In recent years, research on the treatment of antibiotic resistant bacteria has become a boiling point, and significant progress has been achieved in therapy. However, this research is mostly focused on interfering with the resistance of the bacteria, and enhancing the therapeutic effect of antibiotics. Strategies include targeting immune response boosters, antimicrobial resistance inhibitors, vaccines, antibodies, microbiota modifications, drug modifications & hybrids (Cattoir and Felden, 2019). The discoveries of non-antibiotic antibacterial drugs (Ma et al., 2022) are also in urgent demand.

Macrophages are a crucial part in our immune system, and they play an important role in infection. In different pathological conditions, macrophages are mainly polarized into two distinct subsets: M1 and M2. They play different roles in the early stage and recovery stage of infection, and M1/M2 macrophage balance polarization indicates the fate of an organ in inflammation. THP-1 is a human leukemia monocyte cell line; it is a classical model to study macrophage cellular functions (Baxter et al., 2020). Protein posttranslational modifications (PTMs) are essential regulation mechanisms of many life processes at the cellular level. Lysine crotonylation (Kcro) of both histone and non-histone proteins is a newly identified PTM. CREB binding protein (CBP)/p300, male absent on the first (MOF) (Liu et al., 2017), and chromodomain Y-like (CDYL) are involved in the catalytic histone crotonylation (Liu et al., 2019); and histone deacetylases (HDACs) work as an eraser of crotonylation (Abu-Zhayia et al., 2019). Lysine crotonylation participates in many physiological and pathological processes; including chromatin remodeling, metabolism, cell cycle, cell differentiation, inflammatory response, and the pathogenesis of a variety diseases, such as acute kidney injury (Wang et al., 2021). *T. gondii* infection inhibited the crotonylation of histone H2B on K12 in porcine alveolar macrophages, which contributes to promoting macrophage proliferation *via* activation of PI3K/Akt signaling pathway (Yang et al., 2021). Some researches revealed the alteration of crotonylation both in pathogen and host, and concluded the importance of lysine crotonylation in the pathological and physiological processes. The alteration of crotonylation in pathogens, such as fungi, bacteria, and viruses, revealed the importance of lysine crotonylation in the microbial pathological processes. The crotonylated proteins of *Trichophyton rubrum* related to fungal pathogenicity were significantly involved in protein translation, as well as metabolic and biosynthetic processes (Xu et al., 2022). The crotonylated proteins of *Streptococcus agalactiae* were also highly enriched in metabolic, cellular processes, and virulence (Chen et al., 2021). Acetyl-CoA synthetase 2 (ACSS2) enhanced histone crotonylation-induced HIV replication and reactivation (Jiang et al., 2018); crotonylation elevated AZD5582-induced noncanonical NF-κB signaling then augmenting HIV latency (Li et al., 2022). However, acetyltransferase and decrotonyltransferase in bacteria may alter the function of host proteins by altering crotonylation; Rab9A and RAP1B in *Brucella* spp. changed the crotonylation of proteins in HEK-293T cells and could promote intracellular bacterial replication (Zhu et al., 2020). Nevertheless, the post-translational modifications in host cells, such as macrophages, when interacting with *Staphylococcus aureus*, especially MRSA, have not been revealed.

In this study, we examined the alteration of lysine pan-crotonylation in THP1 cells after being infected with MRSA through the use of western blot and by performing a proteome-wide identification of lysine crotonylation. We also analyzed the functions and roles of crotonylated proteins, the amino acid motifs of crotonylated sites, and interaction networks of all identified crotonylation proteins. These results form a foundation for future research on the mechanism of PTM regulating host immune response against MRSA infection and provide new therapeutic targeting strategies to the medical pathogenic bacteria.

## Materials and methods

### MRSA and cell culturing condition

MRSA ATCC43300 stored in our lab was grown in Luria-Bertani (LB) medium shaking at 200 rpm at 37°C overnight to reach the logarithmic phase of growth by the next morning. The growth rate of MRSA ATCC43300 was measured at OD600 (OD600 = 0.5, approximately equivalent to 1.5 × 10^8^ colony forming units (CFU)/mL). THP1 cells (a human monocyte cell line) were purchased from Procell Life Science & Technology Co.Ltd (Wuhan, China) then cultured and grown in RPMI 1640 (Gibco) supplied with 10% heat-inactivated fetal calf serum (Hyclone) and 100 U/mL each of penicillin and streptomycin (Gibco) at 37°C in humidified conditions with 5% CO_2_.

### Peripheral blood mononuclear cell isolation

The patient infected solely with MRSA and the age matched healthy volunteer were enrolled for analysis. The study was approved by the Ethics Committee of Daping Hospital, Chongqing, China (No#112) and informed written consent was signed prior to enrollment. Peripheral blood from patient and healthy control were collected in EDTA tubes then diluted 3 volumes with 1X PBS (PH7.2, 0.01M #20012027, Gibco). PBMCs were isolated according to manufacturer instructions by centrifugation at 1500 g for 30 min on a Ficoll-Paque PREMIUM (17-5442-02, GE). The phase containing the white blood cells was obtained then washed three times with 1X PBS (PH7.2). The PBMCs were used for western blotting analysis directly.

### Western blot analysis

**Figure 1:**
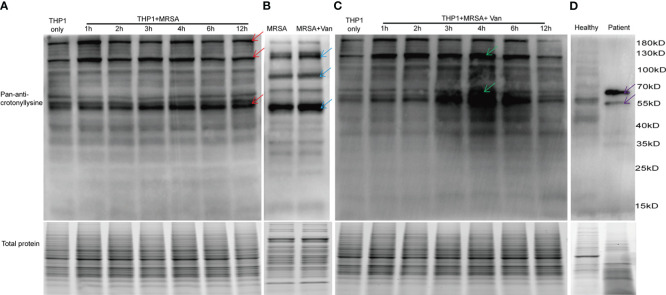
Detection of lysine crotonylation in THP1 cells, MRSA and PBMCs. Western blot analysis by pan-anti-crotonyllysine antibody. Lysates from **(A)** THP1 cells infected by MRSA for different times, and **(B)** MRSA and MRSA treated with vancomycin for 0.5 hours, **(C)** THP1 cells infected by MRSA for different times then treated with vancomycin for 0.5 hours, **(D)** PBMCs from healthy volunteer and MRSA-infected patients. The arrows show the elevated protein bands. Equal loading of total protein was verified using staining free gel.

For western blot analysis of **Figure 1B**, MRSA ATCC43300 8× 10^9^ CFU samples were treated with 2mg/mL vancomycin (Bioss, D21028) or 1X PBS (PH7.2) for 0.5 hours; The samples used for **Figures 1A, C** were prepared from THP1 cells. THP1 cells cultured without penicillin and streptomycin were infected with 0.5 multiplicity of infection (MOI) MRSA ATCC43300 for 1, 2, 3, 4, 6, 12 hours (**Figure 1A**). For **Figure 1C**, the same number and cultured condition of THP1 cells were infected with 0.5 MOI MRSA for 1, 2, 3, 4, 6, 12 hours, washed three times with PBS (PH7.2) and treated with 2mg/mL vancomycin for 0.5 hours. THP1 cells without any treatment were used as control. For **Figure 1D**, the PBMCs from the healthy control and MRSA-infected patient were directly used for protein extraction. Briefly, MRSA, THP1 and PBMCs samples were washed three times with 1X PBS (PH7.2) then the total protein was extracted by radio-immunoprecipitation assay (RIPA) buffer (Beyotime Biotechnology, Shanghai, China) and the concentrations were detected by a bicinchoninic acid (BCA) protein assay kit (Beyotime Biotechnology, Shanghai, China). Equal amounts of protein (20 μg) were separated by sodium dodecyl sulfate-polyacrylamide gel electrophoresis (SDS-PAGE) and transferred onto polyvinylidene fluoride (PVDF) membranes (Millipore, Billerica, MA, USA). After non-specific binding sites were blocked by 1 hour incubation with QuickBlock™ Western reagent (Beyotime Biotechnology, Shanghai, China) at room temperature, the membranes were exposed to primary mouse anti-crotonyllysine antibodies (PTM-502, PTM Bio) at 4°C for overnight. PVDF membranes were then washed with tris-buffered saline (TBS) (PH7.6) with 0.1% Tween-20 three times and incubated with the horseradish peroxidase-conjugated secondary antibodies (#7076S, CST) for 2 h at room temperature. The bands were visualized by using ChemiDoc Touch Imaging System (Bio-Rad, CA, USA) with SuperSignal West Femto Maximum (#34095, ThermoFisher Scientific).

### Protein extraction and trypsin digestion for proteome-wide experiment

About 5×10^7^ THP1 cells infected with 0.5 MOI MRSA for 3 hours and washed three times with 1XPBS (PH7.2) then incubated with 2mg/mL vancomycin for 0.5 hours and the untreated control cells were sonicated three times on ice using a high intensity ultrasonic processor (Scientz) in lysis buffer (8 M urea, 1% protease inhibitor cocktail, 3 μM trichostatin A (TSA) and 50 mM nicotinamide (NAM)). The samples were centrifugated at 12,000g at 4°C for 10 min to remove the cell debris. The supernatants were collected and the protein concentrations were also determined with BCA kit (Beyotime Biotechnology, China) according to the manufacturer’s instructions. Then the protein samples were reduced with 5 mM dithiothreitol for 30 min at 56°C and alkylated with 11 mM iodoacetamide for 15 min at room temperature in darkness. The samples were diluted by 100 mM triethylammonium bicarbonate (TEAB) to a urea concentration less than 2 M. Then trypsin was added at 1:50 trypsin-to-protein mass ratio for the first digestion overnight and 1:100 trypsin-to-protein mass ratio for a second digestion for another 4 hours. Finally, the peptide samples were desalted by C18 SPE column.

### Affinity enrichment for crotonylated peptides

To enrich modified peptides, tryptic peptides were dissolved in NETN buffer (100 mM NaCl, 1 mM EDTA, 50 mM Tris-HCl, 0.5% NP-40, pH 8.0) then incubated with pre-washed mouse anti-crotonyllysine antibody beads (#PT502, PTM Bio) at 4°C overnight with gentle shaking. The next morning, the beads were washed for four times with NETN buffer and three times with ddH_2_O. The bound peptides were eluted from the beads with 0.1% trifluoroacetic acid for three times and the eluted fractions were collected and then vacuum-dried. The collected peptides were desalted with C18 ZipTips (Millipore) and used for liquid chromatography-tandem mass spectrometry (LC-MS)/MS analysis.

#### LC-MS/MS analysis

The total tryptic peptides and the crotonylated enriched peptides were dissolved in solvent A with 0.1% formic acid (#56302-50, Sigma-Aldrich), 2% acetonitrile (#A998–4, Fisher) and loaded onto a home-made reversed-phase analytical column (25-cm length, 75/100 μm i.d.) then isolated with a gradient from 6% to 24% solvent B (0.1% formic acid in acetonitrile) for 70 minutes, 24% to 35% for 14 minutes, and climbing to 80% for 3 minutes twice. All the samples were managed at a constant flow rate of 450 nL/min on a nanoElute ultra-high-performance liquid chromatography (UHPLC) system (Bruker Daltonics). Peptides were injected into capillary source and followed by the timsTOF Pro (Bruker Daltonics) mass spectrometry. The electrospray voltage was 1.60 kV. Precursors and fragments were administrated at the TOF detector, with a MS/MS scan range from 100 to 1700 m/z. The timsTOF Pro was set in parallel accumulation serial fragmentation (PASEF) mode. Precursors with charge states 0 to 5 were used for fragmentation, and 10 PASEF-MS/MS scans were acquired per cycle.

### Enrichment of gene ontology and kyoto encyclopedia of genes and genomes analysis

All the different expressions or modified proteins were classified by Go Ontology (GO) annotation into three categories: biological processes, cellular compartment, and molecular function. For each category, a two-tailed Fisher’s exact test was used to analyze the enrichment of the differentially expressed proteins. Kyoto Encyclopedia of Genes and Genomes (KEGG) database was used for enrichment of pathway analysis. These pathways were classified into hierarchical categories according to the KEGG website. Enrichment of protein domain analysis: For each category of proteins, InterPro (The Integrated Resource of Protein Domains and Functional Sites) database was applied to test the enrichment of the differentially expressed proteins and a two-tailed Fisher’s exact test was utilized to provide functional analysis of protein sequences by classifying them into families and predicting the presence of domains and important sites. The differentially expressed or modified proteins with a corrected *p*-value < 0.05 was considered significant for all the analyses.

### Enrichment-based clustering

Hierarchical clustering analysis is based on differentially modified proteins functional classification (GO and Domain). Proteins for each category were obtained after enrichment along with their *P* values (*P <*0.05). This filtered *P* value matrix was transformed by the function x = −log10 (P value). Finally, these x values were z-transformed for the functional category. These z scores analysis was further clustered by one-way hierarchical clustering in Genesis. Cluster component analysis was showed by a heat map using the “heatmap 2” R-package. Identified proteins domain functional description was annotated by InterProScan (a sequence analysis application) based on protein sequence alignment method, and the InterPro domain database (http://www.ebi.ac.uk/interpro/).

### Subcellular localization

The major components of eukaryotic cells include the nucleus, cytoplasm, extracellular space, mitochondria, endoplasmic reticulum (ER), Golgi apparatus, peroxisome, cytoskeleton, nuclear matrix, and ribosomes. Wolfpsort PSORT II, subcellular localization predication software, was used to predict subcellular localization of eukaryotic cells.

### Motif analysis

MoMo (motif-x algorithm) software was applied to mark the sequence patterns surrounding the Kcro and the sequences constituted with amino acids in specific positions of modified-21-mers (10 amino acids upstream and downstream of the site) in all crotonylated protein sequences. All the different database protein sequences were used as a background database parameter. Minimum number of modified occurrences was set to 20. Emulated original motif-x was marked, and other parameters were set as default. The heat map of motif analysis was generated using R based heatmap package.

### Protein-protein interaction network

All differentially crotonylated proteins were analyzed using STRING database version 11.0 for protein-protein interactions (PPIs). PPIs network was visualized by Cytoscape (3.7.2) software, then some different expression proteins were chosen for analysis functions using the Molecular Complex Detection (MCODE) algorithm.

## Results

### Protein lysine crotonylation changed in THP1 cells after infected by MRSA

To investigate the role of protein lysine crotonylations in host cells confronted with MDR bacterial infections, protein lysates of lysine crotonylations were investigated by western blot in THP1 cells infected by MRSA for 1, 2, 3, 4, 6, and 12 hours. Significant elevations of lysine crotonylation were observed in THP1 cells infected with MRSA for different times compare to uninfected control cells, especially in protein bands around 190 KDa, 120KDa, and 55KDa (**Figure 1A** upper panel, showed by red arrows). Equal loading of total protein was verified using staining free gel in **Figure 1A** lower panel. We attempted to elucidate whether lysine crotonylation levels exist in MRSA or vancomycin-treated samples. Then lysine crotonylation profiles of these samples were tested. Lysine crotonylation was detectable in MRSA bacteria. The increased crotonylated proteins were mainly around 125, 90 and 50 KDa after MRSA treated with vancomycin for 30 minutes (**Figure 1B**, showed by blue arrows). Research showed the alteration of lysine crotonylation also influences the functions of microorganism, such as *Streptomyces roseosporus (* Xu et al., 2022 *)*, *Streptococcus agalactiae* and host cells. To figure out whether the changed proteins of bacteria influenced the protein lysine crotonylations of host cells, the same number of THP1 cells with MRSA infection was further treated by vancomycin for 0.5 hours. Interestingly, we observed a significant increase in lysine crotonylation in these MRSA-infected THP1 cells treated by vancomycin, especially protein bands around 120KDa and 55KDa (**Figure 1C**, showed by green arrows); and the alteration of lysine crotonylation in these vancomycin treated cells for 3, 4, 6 hours was elevated more than the infected cells alone (**Figures 1A, B**). Lysine crotonylation profiles of uninfected THP-1 and uninfected THP-1 cells treated with Vancomycin showed no remarkable difference (**Supplemental Figure 1**). These results suggested that vancomycin treatment could enhance the alteration of lysine crotonylations of MRSA-infected cells. To examine whether lysine crotonylation levels are elevated in human samples as well, we tested lysine crotonylation levels in PBMCs from MRSA-infected patient and a healthy volunteer. Consistent with the observations in MRSA-infected THP1, elevated levels of lysine crotonylation of proteins around 60 and 55 KDa were visible in PBMCs from MRSA-infected individuals relative to an age-matched healthy volunteer (**Figure 1D**, showed by purple arrows). Together, these findings demonstrated that the lysine crotonylation profiles of THP1 cells and bacteria were different and lysine crotonylation of proteins were partially elevated in MRSA-infected THP1, especially when combined with vancomycin treatment.

### Identification of the global differentially regulated proteins in MRSA infected THP1 cells with vancomycin treatment

**Figure 2:**
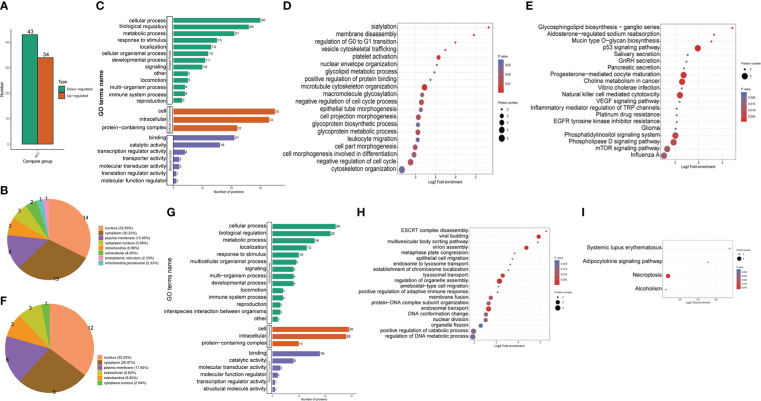
Proteomic identification and bioinformatics analysis of DEPs in THP1 cells in response to MRSA infection combined with vancomycin treatment vs. control cells. **(A)** The number of differentially expressed proteins compared the THP1 cells infected by MRSA for 3 hours then treated by vancomycin for 0.5 hours with control cells. Down-regulated proteins, **(B)** Pie chart of the percentage and number, **(C)** GO (Gene Ontology) pathway enrichment analysis, **(D)** Biological process (BP) category analysis, **(E)** KEGG pathway enrichment analysis. Up-regulated proteins, **(F)** Pie chart of the percentage and number, **(G)** GO pathway enrichment analysis, **(H)** Biological process (BP) category analysis, **(I)** KEGG pathway enrichment analysis.

As the lysine crotonylation profile of MRSA bacteria was detectable from the western blot result, we administrated the infected cells treated with vancomycin in order to remove the influence of bacteria for further proteomic analysis. To validate the differential expression of proteins, lysates from THP1 control cells or THP1 cells infected with MRSA for 3 hours combined with vancomycin administrated for 0.5 hours were used for LC-MS/MS analysis. Our analysis identified 6,018 proteins in all THP1 cells, of which 5,063 proteins were quantifiable (**Supplemental Figure 2**). By comparing MRSA-infected THP1 treated with vancomycin to control THP1 cells, 34 and 43 proteins were found to be up regulated and down regulated, respectively (**Figure 2A**). The differentially down-regulated proteins in MRSA-infected THP1 compared with the uninfected control cells were detected in the nucleus (32.56%), cytoplasm (30.23%), plasma membrane (13.95%), and mitochondria (6.98%) (**Figure 2B**). According to the GO enrichment analysis (**Figure 2C**), biological process (BP) category, 30, 26, 21 and 15 down-regulated proteins were identified in cellular process, biological regulation, metabolic process and as a response to stimulus, respectively. For the cellular component (CC) category, 35, 33, and 22 down-regulated proteins were enriched in cell, intracellular, and protein-containing complex, respectively. 21, 16, and 4 down-regulated proteins were focused in binding, catalytic activity, and transcription regulator activity, respectively, for molecular function (MF) analysis. Differentially down-regulated proteins in biological processes participated in various biological processes, including “negative regulation of cell cycle”, “microtubule cytoskeleton organization”, “cell projection morphogenesis”, and “glycoprotein metabolic process” (**Figure 2D**). KEGG annotation indicated that down-regulated proteins were enriched in “P53 signaling pathway”, “NK cell mediated cytotoxicity”, “phosphatidylinositol signaling system”, “phospholipase D signaling system” and “mTOR signaling pathway” (**Figure 2E**). The results of down regulated proteins of host cells suggested hijacked conditions in response to MRSA infection.

The differentially up-regulated proteins in MRSA-infected THP1 compared with uninfected control cells were predicted to be located in the nucleus (35.29%), cytoplasm (26.47%), plasma membrane (17.65%), and mitochondria (8.82%) (**Figure 2F**). According to the GO enrichment analysis, the biological process (BP) category, 24, 22, 16 and 13 up-regulated proteins were identified in cellular process, biological regulation, metabolic process, and localization, respectively. For the cellular component (CC) category, 29, 28, and 10 up-regulated proteins were enriched in cell, intracellular, and protein-containing complex, respectively. 18, 8, and 3 up-regulated proteins were enriched in binding, catalytic activity, and molecular transducer activity in molecular function category, respectively (**Figure 2G**). With respect to biological processes, viral budding, virion assembly, lysosomal transport, regulation of organelle assembly, and endosomal transport were the most significantly enriched items (**Figure 2H**). The KEGG pathway enrichment analysis showed that a majority of the up-regulated proteins were associated with necroptosis and adipocytokine signaling pathway (**Figure 2I**). The up-regulated proteins of THP1 cells suggested that the host cells instinctively initiated the innate immune response for bacterial clearance.

### Identification of crotonylated proteins and sites in MRSA infected THP1 cells

**Figure 3:**
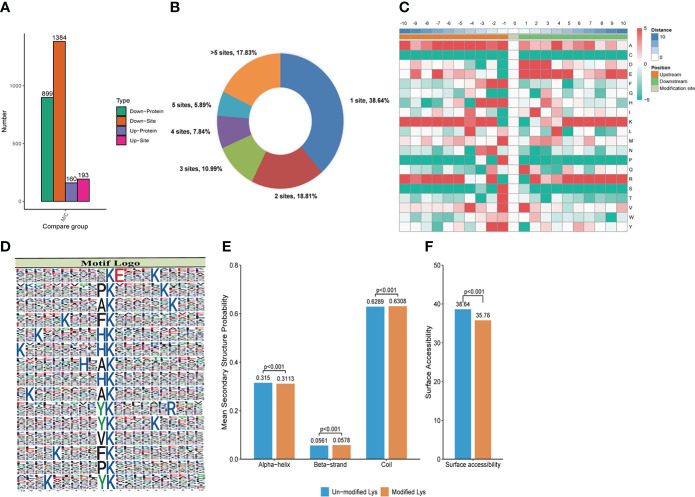
Crotonylation modifications identified in MRSA infected THP1 cells treated with vancomycin vs. control cells. **(A)** The number of differentially crotonylated proteins between the THP1 cells infected by MRSA for 3 hours then treated by vancomycin for 0.5 hours and control cells. **(B)** Pie chart showed the distribution of the number of identified Kcro sites per protein. **(C)** Heat map of the different types of amino acids at positions −10 to +10 from the crotonylated lysine residue. Red and green colors denote high and low frequency, respectively. **(D)** Sequence motif logos, motif, motif score and fold increase showing the crotonylation sites detected in proteins. **(E)** Probabilities of lysine crotonylation in diferent protein secondary structures (alpha helix, beta-strand and disordered coil). **(F)** Predicted surface accessibility of crotonylation sites.

In order to qualify the peptides that were acquired for further analysis, the length of peptides were detected. The results indicated that most of the peptides were formed of 7-20 amino acids, consistent with the rule of trypsin digestion (**Supplemental Figure 3A**). Compared with normal THP1, a total of 3,584 proteins were identified in the MRSA infected THP1, and 2,673 of them were quantifiable (**Supplemental Figure 3B**). To get more insightful information in lysine crotonylation in MRSA infected macrophages, LC-MS/MS and bioinformatic analysis were performed to identify the enriched crotonylated peptides and sites. Lysine-crotonylated peptides were enriched by immuno-affinity using the protein extracted from MRSA-infected THP1 and control cells. After the cells were infected with MRSA and combined with vancomycin treatment, there were 899 proteins and 1384 sites that were down regulated, and 160 proteins and 193 sites that were up-regulated in macrophages (**Figure 3A**). In summary, 38.64%, 18.81%, 10.99%, 7.84%, 5.89%, and 17.83% of total differently modified proteins showed 1, 2, 3, 4, and greater than 5 altered lysine-crotonylated sites, respectively (**Figure 3B**).

### Motif analysis of crotonylated sites in MRSA infected THP1 cells

MoMo software was applied to study the crotonylated sites from 10 amino acids upstream to 10 amino acids downstream the flanking sequences. The frequency of lysine (K) residue was found highly at -10 to -5, -3, -2 and 5 to 10 positions, alanine (A) residue was enriched at the positions -10, -7 to -3, -1 and 4, glutamic acid (E) residue was enriched at -7, 1 to 5, and 9 positions, arginine (R) residue was enriched at -10, -8 to -5, -2, 2, and 5 to 10 positions. The red color represents an amino acid that is significantly enriched near the modification site, while the green color indicates an amino acid that is significantly reduced near the modification site (**Figure 3C**). We also analyzed the position-specific crotonylation frequencies of lysine residues. In all, 82 motifs were identified (**Supplemental Table 1**). These motifs exhibited different abundances and showed that FDK_cro_ was significantly enriched. The motifs of K_cro_E***K_cro_, PK_cro_***K_cro_, AK_cro_***K_cro_, K_cro_***FK_cro_, and K_cro_***HK_cro_ (K_cro_ is the crotonylated lysine and * is any amino acid) showed the highest score in **Figure 3D**. These motifs were likely to represent a feature of crotonylation in MRSA-infected THP1 cells combined with vancomycin treatment. For the secondary structure probability of all modified proteins, approximately 31.13%, 5.78%, and 63.08% of the crotonylated sites were located in α-helices, β-strands, and in disordered coils, respectively (**Figure 3E**). The surface accessibility of the crotonylated lysine sites was about 35.78% and the value was close to the un-crotonylated lysine residues (**Figure 3F**). The results suggested that lysine crotonylation likely does not affect the surface properties of crotonylation modified proteins.

### Functional annotation and classification analysis of the down-regulated crotonylated Proteins in THP1 cells

**Figure 4:**
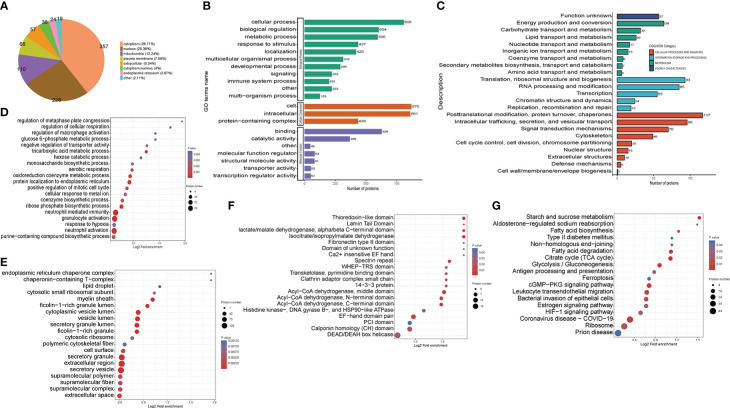
Functional classification of down-regulated crotonylated proteins in THP1 cells infected by MRSA for 3 hours then treated by vancomycin for 0.5 hours compare with control cells. **(A)** Pie chart of the percentage and number of down-regulated crotonylated protein. **(B)** Bar graphs showing GO-based enrichment analysis of the down-regulated crotonylated proteins. **(C)** COG/KOG category analysis. **(D)** Biological Process category analysis. **(E)** Cellular Component category analysis. **(F)** Down regulated domain. **(G)** KEGG pathway analysis.

For better comprehension of the crotonylation altered proteins in MRSA-infected THP1 cells and their corresponding cellular components, biological processes, and molecular functions, we annotated and classified the down-regulated crotonylated proteins. The subcellular location analysis of quantifiable proteins manifested that most of down-regulated crotonylated proteins were predicted to be mainly in the cytoplasm (39.71%), nucleus (25.36%), and mitochondria (12.24%) (**Figure 4A**). GO analysis showed that the down-regulated crotonylated proteins had extensive activity in biological processes and cellular components in THP1 (**Figure 4B**). Clusters of Orthologous Groups/euKaryotic Ortholog Groups (COG/KOG) category analysis of down-regulated modified proteins in THP1 infected cells vs. control cells revealed that the largest modified proteins were functionally clustered in the categories of posttranslational modification and translation/ribosomal structure and biogenesis (**Figure 4C**). The most abundant group of crotonylated proteins in the biological process (BP) category consisted of neutrophil mediated immunity and activity, protein localization to endoplasmic reticulum, and oxidoreduction coenzyme metabolic process (**Figure 4D**). The majority of the crotonylated proteins were associated with cytoplasmic vesicle lumen, secretory granule lumen, and secretory granule/vesicle within the cellular component classification (**Figure 4E**). Characterization of domain of down-regulated crotonylated proteins showed that the modified proteins were found in Acyl-CoA dehydrogenase, isocitrate/isopropylmalate dehydrogenase, and lactate dehydrogenase (**Figure 4F**). Moreover, KEGG pathway analysis revealed that down-regulated crotonylated proteins were significantly enriched in ribosome, fatty acid degradation, TCA cycle, and glycolysis (**Figure 4G**). These observations showed that down-regulated crotonylated proteins have multiple functions, but are mostly located in cytoplasm and mainly play roles in protein posttranslational modification and metabolism in MRSA-infected THP1 cells.

### Subcellular location and functional classification analysis of the up-regulated crotonylated proteins in THP1 cells

**Figure 5:**
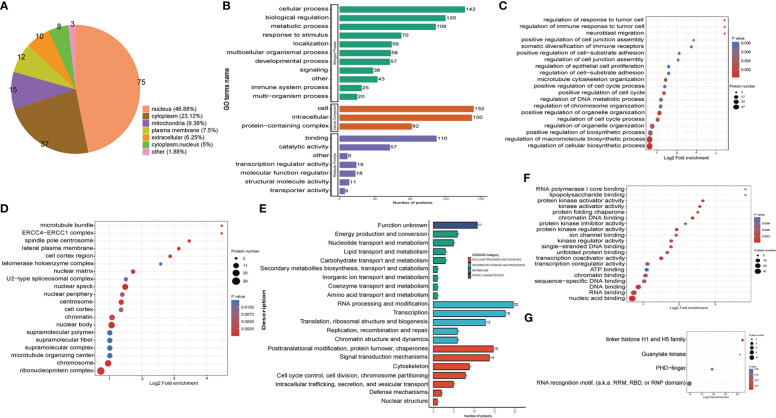
Functional classification of up-regulated crotonylated proteins in THP1 cells infected by MRSA for 3 hours then treated by vancomycin for 0.5 hours compare with control cells. **(A)** Pie chart of the percentage and number of up-regulated crotonylated protein. **(B)** Bar graphs showing GO-based enrichment analysis of the up-regulated crotonylated proteins. **(C)** Biological Process category analysis. **(D)** Cellular Component category analysis. **(E)** COG/KOG category analysis. **(F)** Molecular function analysis. **(G)** Up regulated domain.

The subcellular location analysis of quantifiable proteins manifested that most of the up-regulated crotonylated proteins were predicted to be in the nucleus (46.88%), cytoplasm (23.12%), and mitochondria (9.38%) (**Figure 5A**). GO annotation indicated that for the biological process (BP) category, 142, 120, and 109 were identified in cellular process, biological regulation, and metabolic process. For the cellular component category, proteins 152 and 150 proteins were enriched within the cell and intracellular, respectively. As for the molecular function category, 110 and 57 proteins were associated with binding and catalytic activity (**Figure 5B**). In terms of up-regulated crotonylated proteins, the significant biological processes were “regulation of cellular biosynthetic process”, “regulation of macromolecule biosynthetic process”, and “positive regulation of biosynthetic process” (**Figure 5C**). In the cellular component category, the greatest number of up-regulated proteins in terms of abundance was found in the ribonucleoprotein complex, chromosomes, microtubule organizing center, and nuclear speck (**Figure 5D**). Clusters of Orthologous Groups revealed that the largest modified proteins were functionally clustered in the categories of RNA processing and modification, transcription, signal transduction mechanisms, and translation/ribosomal structure and biogenesis (**Figure 5E**). These up-modified proteins were involved in nucleic acid, RNA and DNA binding (**Figure 5F**). Specifically, the domain of up-regulated proteins mainly focused on RNA recognition motif and linker histone H1 and H5 family (**Figure 5G**). These observations showed that crotonylation was enriched in several types of proteins and pathways, suggesting a pivotal role of lysine crotonylation in the nucleus, especially RNA regulation.

### Characteristics of clustering analysis of altered crotonylated proteins related to THP1 response against MRSA infection

**Figure 6:**
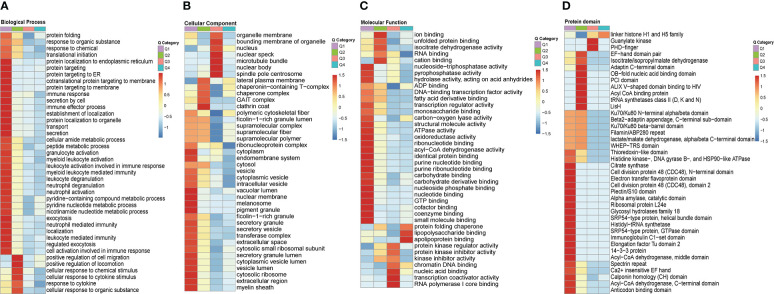
Clustering analysis of identified up-regulted crotonylated proteins and functional characterization of crotonylated proteins. Fold changes of modified crotonylation sites in MRSA-infected THP1 cells vs. control cells were classified into four classes; Q4 (>2.0, up-regulated 2.0 fold),Q3(1.5-2.0, up-regulated 1.5-2.0 fold), Q2(0.5-0.667, down-regulated 1.5-2.0 fold) and Q1(<0.5, down-regulated 2.0 fold). Heat maps showing the results of a cluster analysis of **(A)** Biological Process category analysis, **(B)** Cellular Component category analysis, **(C)** Molecular Function category analysis from the GO term, and **(D)** protein domain analyse.

In order to gain a deeper understanding of the biological function and process of total host crotonylated proteins against MRSA infection, heat maps of the clustering analysis (GO and protein domain) were generated to present the correlations of functions and processes with differentially crotonylated sites. Fold changes of modified crotonylation sites in MRSA-infected THP1 cells *VS.* control cells were classified into four classes; 64, 129, 641, and 743 proteins represent an up-regulation of more than 2.0 fold (Q4, >2.0), up-regulation of 1.5-2.0 fold (Q3, 1.5-2.0), down-regulation of 1.5-2.0 fold (Q2, 0.5-0.667) and down-regulation more than 2.0 fold (Q1, <0.5) crotonylation sites, respectively (**Supplemental Figure 4A**). In the biological process category, proteins of down-regulated crotonylation sites greater than 2.0 fold (Q1), mainly were enriched in “protein localization to endoplamic reticulum”, “cotranslational protein targeting to membrane”, “protein targeting to membrane”, “immune response”, and “leukocyte mediated immunity”. Proteins of down-regulated crotonylation sites between 1.5-2.0 fold (Q2), differentially crotonylated proteins were enriched in “positive regulation of cell migration”, “positive regulation of locomotion”, “cellular response to chemical stimulus”, and “response to cytokine” (**Figure 6A**). In the cellular component category, proteins in Q1 mainly were involved in “cytoplasm”, “endomembrane system”, “nuclear membrane”, and “cytosolic ribosome”. As for the proteins in Q2, differentially crotonylated proteins mainly participated in “GAIT complex”, “chaperone complex” and “lateral plasma membrane”. Proteins of up-regulated crotonylation sites between 1.5-2.0 fold (Q3) were enriched in “nuclear body” and “nuclear speck” (**Figure 6B**). In the molecular function category, proteins in Q1 played roles in “nucleoside-triphosphatase activity”, “pyrophosphatase activity”, “acyl-CoA dehydrogenase activity”, and “coenzyme binding”. Proteins in Q2 were enriched in “ion binding”, “cation binding”, and “isocitrate dehydrogenase activity”. Proteins in Q3 showed roles in “nucleic acid binding”, “transcription coactivator activity” and “RNA polymerase I core binding”. Interestingly, only in proteins of up-regulated crotonylation sites greater than 2 fold (Q4) were enriched in “apolipoprotein binding”, “lipopolysaccharide binding” and “protein folding chaperone” (**Figure 6C**). All the protein domains of differently modified crotonylation sites in category Q1-Q4 were predicted to participate in “Acyl-CoA dehydrogenase”, “Acyl CoA binding protein”, “Guanylate kinase” and “linker histone H1 and H5 family”, respectively (**Figure 6D**).

### Protein-protein interaction networks of the crotonylated proteins in MRSA-infected THP1 cells

**Figure 7:**
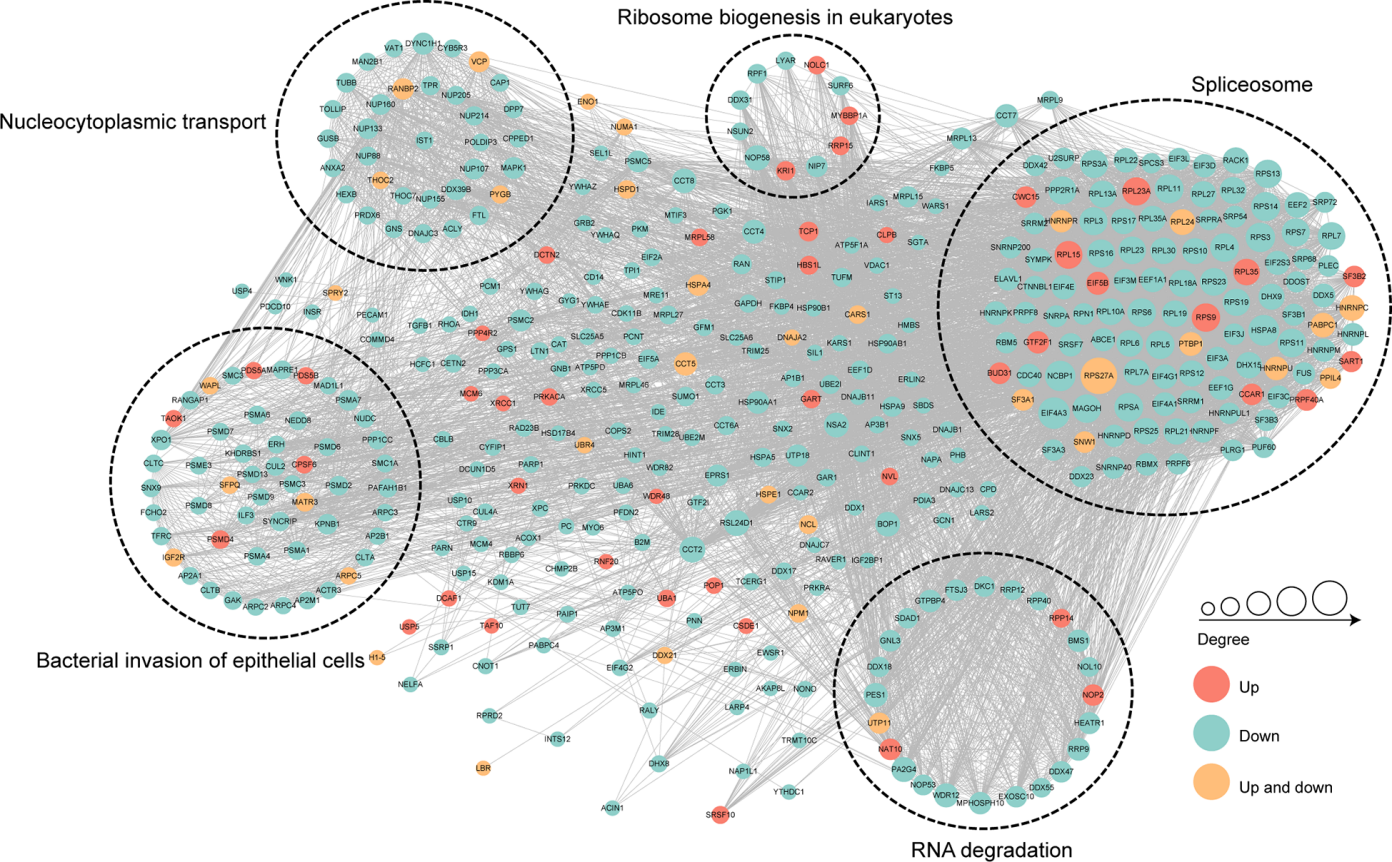
Protein-protein interaction network of the differentially crotonylated proteins. Protein-protein interaction network. Red nodes means up-regulated crotonylated proteins, blue nodes means down-regulated crotonylated proteins. Orange nodes means up and down regulated crotonylated proteins. The degree of nodes means the number of interacting proteins, the more protein nodes interacting with the protein, the node of this protein is bigger.

To further identify the cellular process regulated through crotonylation in MRSA infected THP1 cells, PPI network of the Kcro proteins was studied. A total of 7,900 pairs of protein-protein mapping to the protein interaction database showed the different cellular functions of crotonylated proteins in MRSA infected macrophages. Almost all down-regulated crotonylated proteins were mainly clustered into five groups including the spliceosome, bacterial invasion of epithelial cells, RNA degradation, nucleocytoplasmic transport, and ribosome biogenesis in eukaryotes (**Figure 7**). Among these up-regulated crotonylated proteins in PPI analysis, TAOK1 (Pennemann et al., 2021) and SART1 (Teng et al., 2021) reported the effects on type-I interferon induction and exerted the anti-viral activity. These proteins may play an important role in bacterial infection. The PPI network analysis indicated that lysine malonylation mainly occurs within the spliceosome, which mediated splicing of the precursor mRNA, involving intron removal and exon ligation.

## Discussion

The death rate of infection-triggered sepsis is still very high worldwide (World Sepsis Congress April 21–22, 2021). MDR bacterial infections, such as MRSA mutant strains, spread quickly among patients in hospitals (Jernigan et al., 2020), and frequently arouse severe and overwhelming disease among healthy people (Wu et al., 2019). As bacteria that are commonly encountered in clinical settings, MRSA has been listed as a Priority 2 (Urgency “High”) pathogen for research and development of new therapeutic approaches by WHO (Duval et al., 2019). Research on protein PTM could provide new insights for developing therapeutics against the disease *via* targeting specific steps of the protein post-translational pathway (Proulx et al., 2020). However, the regulatory mechanism of the lysine PTM of human macrophages infected with MRSA is less clear. To investigate the role of lysine crotonylation in the regulation of THP1 cells response to MRSA infection, we performed the first global proteomic survey of lysine crotonylation in the cells. In the present study, we analyzed crotonylation patterns in MRSA-infected THP1 cells and uninfected control cells. Our findings demonstrated that the lysine crotonylation profiles exist widely in eukaryotes and prokaryotes and were different between the bacteria and human monocyte cell lines, especially in the response of MRSA to antibiotic stress.

In the current study, we analyzed globally differentially regulated proteins including the down-regulated proteins that are mainly involved in mTOR signaling pathway, NK cell mediated cytotoxicity, P53 signaling pathway, glycoprotein metabolic process, and phosphatidylinositol signaling system. mTOR complex 1 signaling protected macrophages from mycobacterium-triggered cell death by elevating infection-induced energy metabolism (Pagan et al., 2022). The infection of *Trueperella pyogenes* induced the expression of mTOR and subsequently inhibited the autophagy of host liver cell. However, inhibition of mTOR signaling induced autophagy and reduced bacterial viability in RAW264.7 macrophages (Huang et al., 2019). Viral transmissible gastroenteritis infection activated p53 signaling pathway to induce apoptosis of host cells (Ding et al., 2019). The reduced p53/mTOR signal pathway increased apoptosis and autophagy of THP1 cells. In addition, natural killer cells were critical for host defenses against pathogens (Dong et al., 2019). The responses of NK cells included cytotoxicity, activating receptors, and cytokines production (Chen et al., 2020). The down-regulated cytotoxicity pathway of NK cells reduced bacterial clearance. Macrophages enhanced secretion inflammatory mediators such as TNF-α, IL-6, RANTES, and G-CSF depending on sphingosine kinase (SPK), phosphoinositide-specific phospholipase C (PI-PLC), conventional protein kinase C (cPKC), ERK1/2 and phosphatidylinositol 3-kinase (PI3K) activity (Yadav et al., 2006). The glycerolipid metabolism pathway, particularly those involving the components of glycerolipid/free fatty acid (GL/FFA) cycle, was strongly associated with MRSA infection (Sharma-Kuinkel et al., 2013). The down-regulated metabolic pathway revealed the vulnerable response of host cells to pathogenic infection. We also found that the up-regulated proteins were enriched in cell, intracellular, protein-containing complex, binding, catalytic activity, molecular transducer activity, necroptosis, and adipocytokine signaling pathway. Viral infection or induction of DNA damage activates p53 and p21, which in turn causes a large number of cell cycle genes to decrease, resulting in cell cycle arrest (Engeland, 2022). Bacterial factors that inhibit host DNA synthesis and increase cell cycle arrest could be part of an important strategy used by intracellular pathogens to manipulate the host cell cycle and promote bacterial replication (Sol et al., 2019). Brucella (a facultative intracellular bacterium) infection induced macrophage death depends on T4SS secretion activity (Tian et al., 2020). Up-regulated catalytic activity of special protein may help host cell to eliminate bacteria (Shi et al., 2022). From a model of MRSA infection for 24h murine sepsis, blood samples showed the vancomycin treatment down regulated Toll-like receptor signaling pathway and IL-1β production, but not IL-6 and TNF-α production (Sharma-Kuinkel et al., 2013). This agrees with the fact that vancomycin treatment for a half hour in our cell model showed a weak effect of response of host cells. Taken together, our results suggested that THP1 underwent infection-trigged cell death, autophagy, and reduced cell immune response abilities after MRSA infection.

Numerous proteins of significantly changed Kcro levels in THP1 cells were all dramatically down regulated after MRSA infection combined with antibiotic stress. Therefore, we can infer that MRSA infection inhibits global Kcro modification from MS analysis; but lysine crotonylation of proteins were partially elevated in MRSA-infected THP1 in the same condition analyzed by western blot. In addition, bioinformatics analysis revealed that the down-regulated Kcro proteins were more concentrated in cytoplasm and basically enriched in cell immunity, ribosome, posttranslational modification, intracellular trafficking, secretion, signal transduction mechanisms, and metabolism. More than 90% of these top 50 down-regulated Kcro proteins displayed the size from 135 to 2639 KDa, not around 120 and 55 KDa. Moreover, most of these proteins altered the functions involved in metabolism, such as GPI (Glucose-6-phosphate isomerase), MDH2 (NAD-dependent malate dehydrogenase), and LONP1 (Mitochondrial ATP-dependent protease PIM1/LON). The results suggested that infection and vancomycin treatment altered the condition of host cells.

GO analysis showed that “protein localization to endoplasmic reticulum” pathway decreased during this cell model; and endoplasmic reticulum (ER)-localized Hrd1 expanded TLR4 signal induced inflammation during bacterial infection (Lu et al., 2019). BAG2 ameliorates ER stress-induced cell apoptosis in *M. tuberculosis* -infected macrophages through MAPK/ERK mediated autophagy (Liang et al., 2020). In addition, down-regulated Kcro proteins of “Neutrophil mediated immunity”, “granulocyte activation”, and “neutrophil activation” pathways were significantly reduced. Granulocytes, including neutrophils, are known to play key roles in eliminating pathogens. Human neutrophils mediate infection and immunity through leukocyte immunoglobulin-like receptors (LILRs) (Lewis Marffy and McCarthy, 2020). Neutrophil release of IL-1β and CXCL2 activate defensin Mrgpra2 which were critical for combating *S. aureus* infections (Dong et al., 2022). The present data also suggested that the response of host cells against the infection of MRSA could not be guaranteed. However, 7 crotonylated sites of HSP90B1, Endoplasmic reticulum glucose-regulated protein (GRP94), also were down regulated; HSP90B1 interacts with RhoB and Beclin promotes *Escherichia coli* clearance through inducing LC3 lipidation and autophagosome formation (Miao et al., 2021). Acyl-CoA dehydrogenase (C-terminal, N-terminal and middle domain) was significantly found in down-regulated crotonylated proteins, implying an important role for lysine crotonylation in the protection against bacterial processes. Intracellular crotonyl-CoA controls transcription *via* p300-catalyzed histone crotonylation (Sabari et al., 2015). It was reported that multiple Acyl-CoA dehydrogenase deficiency killed *Mycobacterium tuberculosis (* Beites et al., 2021 *)*. Acyl-CoA dehydrogenase knockout mice were susceptible to H1N1 influenza infection which may be due to increased reliance upon glucose for energy (Shinde et al., 2018). The functions of these proteins in the endoplasmic reticulum and the metabolism proteins for host cells responding to bacterial infection are well known; but the functions of these down-modified crotonylated proteins have not yet been revealed completely. Here, we also observed that crotonylated sites of Histone acetyltransferase type B catalytic subunit (HAT1), Transcription initiation factor TFIID (subunit TAF10), Histone H3 (Lys4) methyltransferase complex and RNA cleavage factor II complex (subunit SWD2), and Histone deacetylase complex (HDAC catalytic component RPD3, SIN3) also presented significant decreases in crotonylation levels. HAT1 regulated histone modifications and was the target of ACE2 (Pinto et al., 2020). SIN3 increased the virulence of lactate-exposed Candida; TAF10 controlled the drug tolerance and virulence of *C. albicans (* de Assis et al., 2022 *)*. Histone deacetylase (HDAC) 1 and 2 complexes regulate both histone acetylation and crotonylation as an eraser *in vivo* (Kelly et al., 2018). Lysine modification of RPD3 involved in the growth, development and virulence of *Beauveria bassiana (* Cai et al., 2018 *)*. These results suggested the depressed effects of protein modifications in THP1 after MRSA infection.

However, the up-regulated Kcro proteins in the present research were mainly located in nucleus, enriched in nuclear body, chromosome, RNA processing and modification, and core histone H2A/H2B/H3/H4; the domains significantly focused on RNA recognition motif and linker histone H1 and H5 family. In addition, a total of 17 up-regulated histone Kcro sites of Histone H1 were identified. Post-translational modifications in H1 have been related to the regulation of chromatin structure, transcriptional activation, and DNA damage response (Andres et al., 2020). Zebrafish histone H2A interacts with RIP2 to induce the expression of antimicrobial genes and MHC related genes (Wu et al., 2019). The crotonylation of histone lysine is specifically enriched at gene promoters and potential enhancers (Tan et al., 2011); this may be suggested as a robust indicator of active promoters in histones. Our results suggested the increased crotonylation activity of histone H1 and core histone complex in THP1 occurs after MRSA infection.

In summary, using high-resolution LC-MS/MS coupled with highly sensitive immune-affinity purification, we investigated the global quantitative proteomics and alteration of lysine crotonylation proteome. We reported the proteome-wide profiling of Kcro in THP1 cells infected with MRSA and identified 899 proteins and 1384 sites were down-regulated, and 160 proteins and 193 sites were up- regulated in macrophages for the first time. The crotonylated down-regulated proteins were mainly located in cytoplasm and it was suggested that they mainly played a role in posttranslational modification. However, the crotonylated up-regulated proteins were mainly located in the nucleus and were significantly involved in modulating chromosome modification and RNA processing. The present data widens the scope of protein crotonylation of human macrophages and provides new insights into the exploration of the physiological regulation of protein crotonylation in host cells against MRSA infection.

## Data availability statement

The datasets presented in this study can be found in online repositories: https://ngdc.cncb.ac.cn/omix/releaseList; Data Accession Number: OMIX002820.

## Ethics statement

The studies involving human participants were reviewed and approved by The Ethics Committee of Daping Hospital, Chongqing, China (No#112). The patients/participants provided their written informed consent to participate in this study. Written informed consent was obtained from the individual(s) for the publication of any potentially identifiable images or data included in this article.

## Author contributions

All authors listed, have performed the experiments, analysis and discussion the data of the work, and approved it for publication.

## Supplementary material

Supplementary Figure 1 Western blot analysis by pan-anti-crotonyllysine antibody of the lysates from uninfected THP-1 and uninfected THP-1 cells treated with Vancomycin.

Supplementary Figure 2 Number of each peptide from proteomic analysis.

Supplementary Figure 3 The feature and number of peptides from crotonylation analysis. (A) The peptide length digested by trypsin. (B) The number of identified proteins.

Supplementary Figure 4 Number of crotonylated sites from regulated peptides.
