# Supplementary material for: Systematic analysis of lysine crotonylation in human macrophages responding to MRSA infection
Source: Front Cell Infect Microbiol. 2023 Feb 8;13:1126350. doi: 10.3389/fcimb.2023.1126350 (PMC9945341; doi:10.3389/fcimb.2023.1126350)
Supplement: Supplementary file 1 [file Table_1.docx]

Table 1 Crotonylated lysine motifs in THP1 cells infected with MRSA.

| **Motif** | **Motif Score** | **Foreground** | | **Background** | | **Fold Increase** |
| --- | --- | --- | --- | --- | --- | --- |
|  |  | **Matches** | **Size** | **Matches** | **Size** |  |
| xxxxxxxxFD_K_xxxxxxxxxx | 24.73 | 29 | 600 | 736 | 146781 | 9.6 |
| xxxxxxxxxW_K_xxxxxxKxxx | 22.79 | 31 | 1533 | 530 | 215213 | 8.2 |
| xxxxRxxxxF_K_xxxxxxxxxx | 23.74 | 41 | 3256 | 541 | 306252 | 7.1 |
| xxxxxxxxFE_K_xxxxxxxxxx | 30.87 | 68 | 1502 | 1364 | 214683 | 7.1 |
| xxxxxxxxHI_K_xxxxxxxxxx | 22.86 | 23 | 872 | 643 | 171688 | 7.0 |
| xxxxxxxxRI_K_xxxxxxxxxx | 30.52 | 49 | 1101 | 1202 | 181484 | 6.7 |
| xxxxxxxxRx_K_Exxxxxxxxx | 21.82 | 25 | 930 | 712 | 173489 | 6.6 |
| xxxxxxxxxY_K_xxxKxxxxxx | 23.41 | 46 | 3215 | 680 | 305711 | 6.4 |
| xxxxxxxxKI_K_xxxxxxxxxx | 32.00 | 92 | 2209 | 1767 | 261488 | 6.2 |
| xxxxxxxxKM_K_xxxxxxxxxx | 27.51 | 79 | 4438 | 1042 | 353182 | 6.0 |
| xxxxxxxxYE_K_xxxxxxxxxx | 26.35 | 33 | 905 | 1089 | 172777 | 5.8 |
| xxxKxxxxxF_K_xxxxxxxxxx | 32.00 | 121 | 11757 | 1256 | 589851 | 4.8 |
| xxxxxxxxxY_K_xxxxxxxKxx | 31.41 | 69 | 10532 | 795 | 567865 | 4.7 |
| xxxxxxxHxA_K_xxxxxxxxxx | 25.28 | 45 | 7360 | 630 | 473411 | 4.6 |
| xxxxxxxFxG_K_xxxxxxxxxx | 26.41 | 46 | 2255 | 1162 | 262650 | 4.6 |
| KxxxxxxxxH_K_xxxxxxxxxx | 29.11 | 55 | 9947 | 695 | 559671 | 4.5 |
| xxxxxxxxRL_K_xxxxxxxxxx | 32.00 | 158 | 5030 | 2687 | 373028 | 4.4 |
| xxxxxxxHxG_K_xxxxxxxxxx | 22.59 | 31 | 1564 | 979 | 216192 | 4.4 |
| xxxxxxxxxH_K_Exxxxxxxxx | 32.00 | 96 | 12663 | 1070 | 604474 | 4.3 |
| xxKxxxxxxY_K_xxxxxxxxxx | 30.07 | 70 | 10869 | 887 | 574571 | 4.2 |
| xxxxxxxxxY_K_xxxxxxRxxx | 23.88 | 38 | 8245 | 555 | 509219 | 4.2 |
| xxxxxxxxxY_K_xxxxKxxxxx | 32.00 | 93 | 12226 | 1104 | 597031 | 4.1 |
| xxxKxxxxxY_K_xxxxxxxxxx | 28.18 | 57 | 10404 | 751 | 566134 | 4.1 |
| xxxxxxxxKL_K_xxxxxxxxxx | 32.00 | 292 | 10347 | 4029 | 565383 | 4.0 |
| xxKxxxxxxH_K_xxxxxxxxxx | 26.55 | 56 | 10055 | 802 | 561354 | 3.9 |
| xxxKxxxxxH_K_xxxxxxxxxx | 25.67 | 45 | 9598 | 658 | 552750 | 3.9 |
| xxxxxxxxxP_K_xxxxRxxxxx | 22.00 | 50 | 6160 | 891 | 424763 | 3.9 |
| xxxxxxxxxE_K_Vxxxxxxxxx | 23.68 | 45 | 645 | 2750 | 149531 | 3.8 |
| xxxxxxxxxY_K_xxxxxxxRxx | 23.33 | 43 | 9207 | 682 | 545121 | 3.7 |
| xxxxxxxxAE_K_xxxxxxxxxx | 19.48 | 36 | 571 | 2502 | 146045 | 3.7 |
| xxxxKxxxxF_K_xxxxxxxxxx | 29.85 | 89 | 11987 | 1235 | 593286 | 3.6 |
| xxxxxxxxxF_K_xxxxKxxxxx | 26.75 | 58 | 9463 | 925 | 550136 | 3.6 |
| xxxxxxxxxG_K_xxxxxxxxKx | 23.49 | 60 | 1624 | 2332 | 218524 | 3.5 |
| xxxxxxxxxE_K_Gxxxxxxxxx | 20.81 | 43 | 849 | 2483 | 171045 | 3.5 |
| KxxxxxxxxP_K_xxxxxxxxxx | 30.75 | 101 | 10970 | 1551 | 576122 | 3.4 |
| xxxxxKxxxF_K_xxxxxxxxxx | 26.06 | 59 | 10463 | 936 | 567070 | 3.4 |
| xxxxxxxxxP_K_xxxxxKxxxx | 31.38 | 110 | 11207 | 1732 | 580378 | 3.3 |
| xxxxxxxxxH_K_xxxxxKxxxx | 24.20 | 52 | 9999 | 881 | 560552 | 3.3 |
| xxxxxxxxxx_K_xExxxxxxxx | 7.23 | 26 | 114 | 4727 | 68156 | 3.3 |
| xxxxxxxxxF_K_Exxxxxxxxx | 27.93 | 109 | 12567 | 1613 | 603404 | 3.2 |
| xxxxxxxxxP_K_xxxxKxxxxx | 32.00 | 141 | 11898 | 2200 | 592051 | 3.2 |
| xxxxxxxxxx_K_xExxRxxxxx | 25.96 | 121 | 9405 | 2298 | 549211 | 3.1 |
| xxxxxxxxxP_K_xxxxxRxxxx | 23.01 | 65 | 9164 | 1258 | 544439 | 3.1 |
| xxxxxxxxxT_K_xxxxxRxxxx | 22.55 | 55 | 7315 | 1135 | 472781 | 3.1 |
| xxxxxxxxxH_K_xxxxxxxxxx | 16.00 | 330 | 4359 | 8530 | 352140 | 3.1 |
| xxxxxxxxxW_K_xxxxxxxxxx | 16.00 | 122 | 1052 | 6793 | 180282 | 3.1 |
| xxxxxxxxxP_K_xxxxxxKxxx | 27.83 | 110 | 11449 | 1890 | 584656 | 3.0 |
| xxxxxxxxxT_K_xxxxxxxRxx | 22.99 | 63 | 7423 | 1351 | 474762 | 3.0 |
| xxxxxxxxxL_K_xxKxxxxxxx | 24.30 | 80 | 2335 | 3115 | 265765 | 2.9 |
| xxxxxxxxxx_K_DxxxxxxxKx | 30.46 | 132 | 11339 | 2388 | 582766 | 2.8 |
| xxxxxxxxxx_K_xxExRxxxxx | 31.75 | 146 | 12133 | 2641 | 595927 | 2.7 |
| xxKxxxxxxA_K_xxxxxxxxxx | 26.02 | 101 | 9892 | 2087 | 558976 | 2.7 |
| xxxxxxxxxV_K_xxxxxxxKxx | 25.11 | 108 | 9706 | 2293 | 555043 | 2.7 |
| xxxxxxxxxT_K_xxxxxxKxxx | 25.19 | 90 | 9553 | 1956 | 552092 | 2.7 |
| xxxxxxxxxY_K_xxxxxxxxxx | 16.00 | 249 | 2584 | 9773 | 275538 | 2.7 |
| xKxxxxxxxA_K_xxxxxxxxxx | 28.26 | 127 | 11097 | 2524 | 578646 | 2.6 |
| xxxxxxxxxA_K_xxxxxxKxxx | 25.71 | 115 | 10647 | 2366 | 570231 | 2.6 |
| xxxxxxxxxS_K_xIxxxxxxxx | 24.25 | 85 | 9791 | 1846 | 556889 | 2.6 |
| xxxxxxxxxx_K_xxDxxKxxxx | 22.56 | 74 | 7497 | 1842 | 476604 | 2.6 |
| xxxxxxxxxx_K_xIxxxxxKxx | 22.43 | 77 | 9284 | 1792 | 546913 | 2.5 |
| xxxxxxxxxM_K_xxxxxxxxxx | 16.00 | 314 | 4029 | 10860 | 343610 | 2.5 |
| xxxxxxxxxF_K_xxxxxxxxxx | 16.00 | 269 | 3169 | 10280 | 305031 | 2.5 |
| xxxxxxxxxx_K_ExxxxxxKxx | 27.15 | 232 | 12458 | 4760 | 601791 | 2.4 |
| xxxxxxxxxx_K_ExxxKxxxxx | 27.24 | 187 | 11636 | 3939 | 588595 | 2.4 |
| xKxxxxxxxS_K_xxxxxxxxxx | 23.92 | 111 | 8356 | 2874 | 512093 | 2.4 |
| xxxxKxxxxx_K_Exxxxxxxxx | 26.14 | 152 | 10799 | 3453 | 573684 | 2.3 |
| xxxxxxxxxC_K_xxxxxxxxxx | 7.07 | 45 | 159 | 9743 | 77899 | 2.3 |
| xxxxxxxxxx_K_Exxxxxxxxx | 9.64 | 84 | 535 | 10877 | 143543 | 2.1 |
| xxxxxxxxxP_K_xxxxxxxxxx | 16.00 | 434 | 4872 | 17159 | 370341 | 1.9 |
| xxxxxxxxxI_K_xxxxxxxxxx | 12.18 | 161 | 806 | 19031 | 168562 | 1.8 |
| xxxxxxxxxD_K_xxxxxxxxxx | 9.97 | 118 | 451 | 19350 | 132666 | 1.8 |
| xxxxxxxxxN_K_xxxxxxxxxx | 16.00 | 316 | 2900 | 19213 | 294751 | 1.7 |
| xxxxxxxxxE_K_xxxxxxxxxx | 14.80 | 174 | 333 | 35417 | 113316 | 1.7 |
| xxxxxxxxxx_K_Dxxxxxxxxx | 16.00 | 525 | 6685 | 22505 | 447268 | 1.6 |
| xxxxxxxxxT_K_xxxxxxxxxx | 16.00 | 451 | 6110 | 19110 | 423872 | 1.6 |
| xxxxxxxxxQ_K_xxxxxxxxxx | 16.00 | 459 | 3715 | 26498 | 332750 | 1.6 |
| xxxxxxxxxG_K_xxxxxxxxxx | 16.00 | 333 | 1434 | 31835 | 213319 | 1.6 |
| xxxxxxxxxA_K_xxxxxxxxxx | 16.00 | 575 | 7260 | 24378 | 471646 | 1.5 |
| xxxxxxxxxL_K_xxxxxxxxxx | 16.00 | 493 | 2117 | 41197 | 259721 | 1.5 |
| xxxxxxxxxV_K_xxxxxxxxxx | 16.00 | 743 | 9099 | 31088 | 543181 | 1.4 |
| xxxxxxxxxx_K_xxxxxxxKxx | 16.00 | 710 | 8207 | 32060 | 508664 | 1.4 |
| xxxxxxxxxS_K_xxxxxxxxxx | 16.00 | 629 | 5659 | 31734 | 404762 | 1.4 |
