# Supplementary figures and images for: Systematic analysis of lysine crotonylation in human macrophages responding to MRSA infection
Source: Front Cell Infect Microbiol. 2023 Feb 8;13:1126350. doi: 10.3389/fcimb.2023.1126350 (PMC9945341; doi:10.3389/fcimb.2023.1126350)

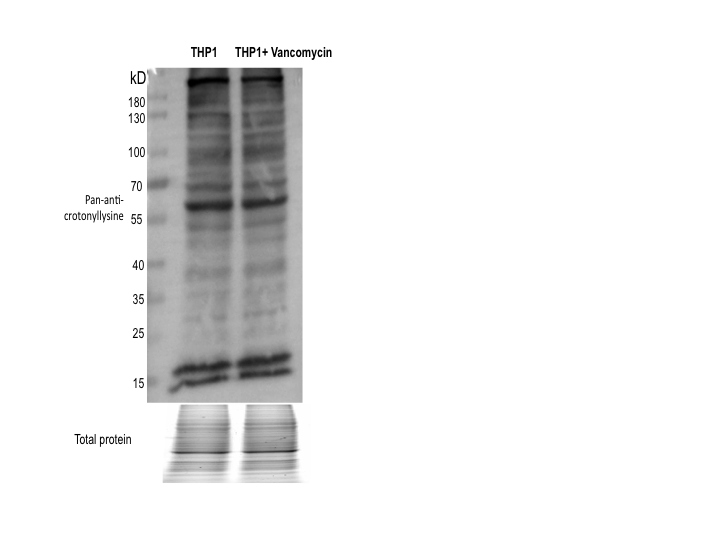

Supplement: Supplementary Figure 1 — Western blot analysis by pan-anti-crotonyllysine antibody of the lysates from uninfected THP-1 and uninfected THP-1 cells treated with Vancomycin. [file Image_1.tiff]

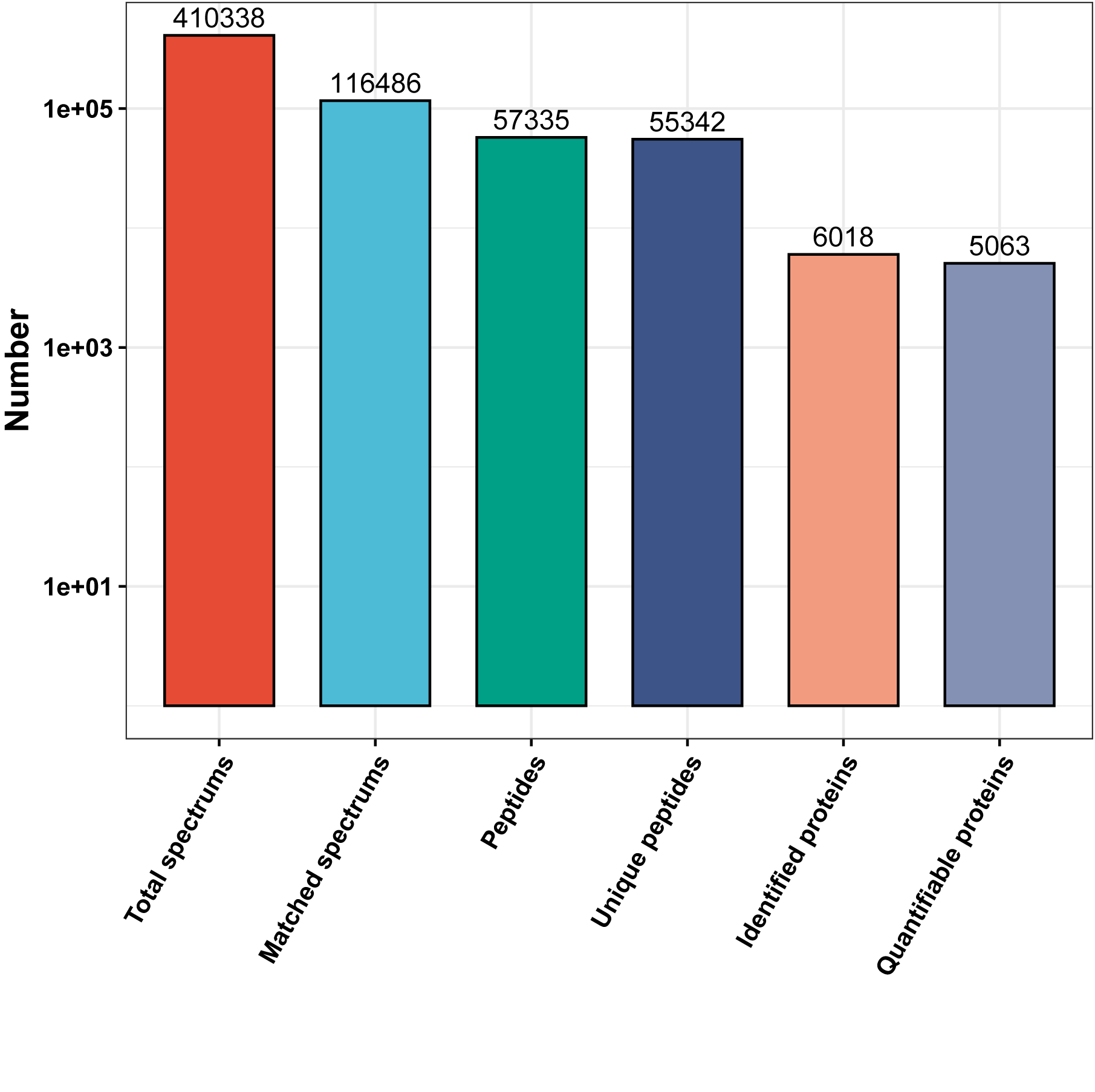

Supplement: Supplementary Figure 2 — Number of each peptide from proteomic analysis. [file Image_2.tif]

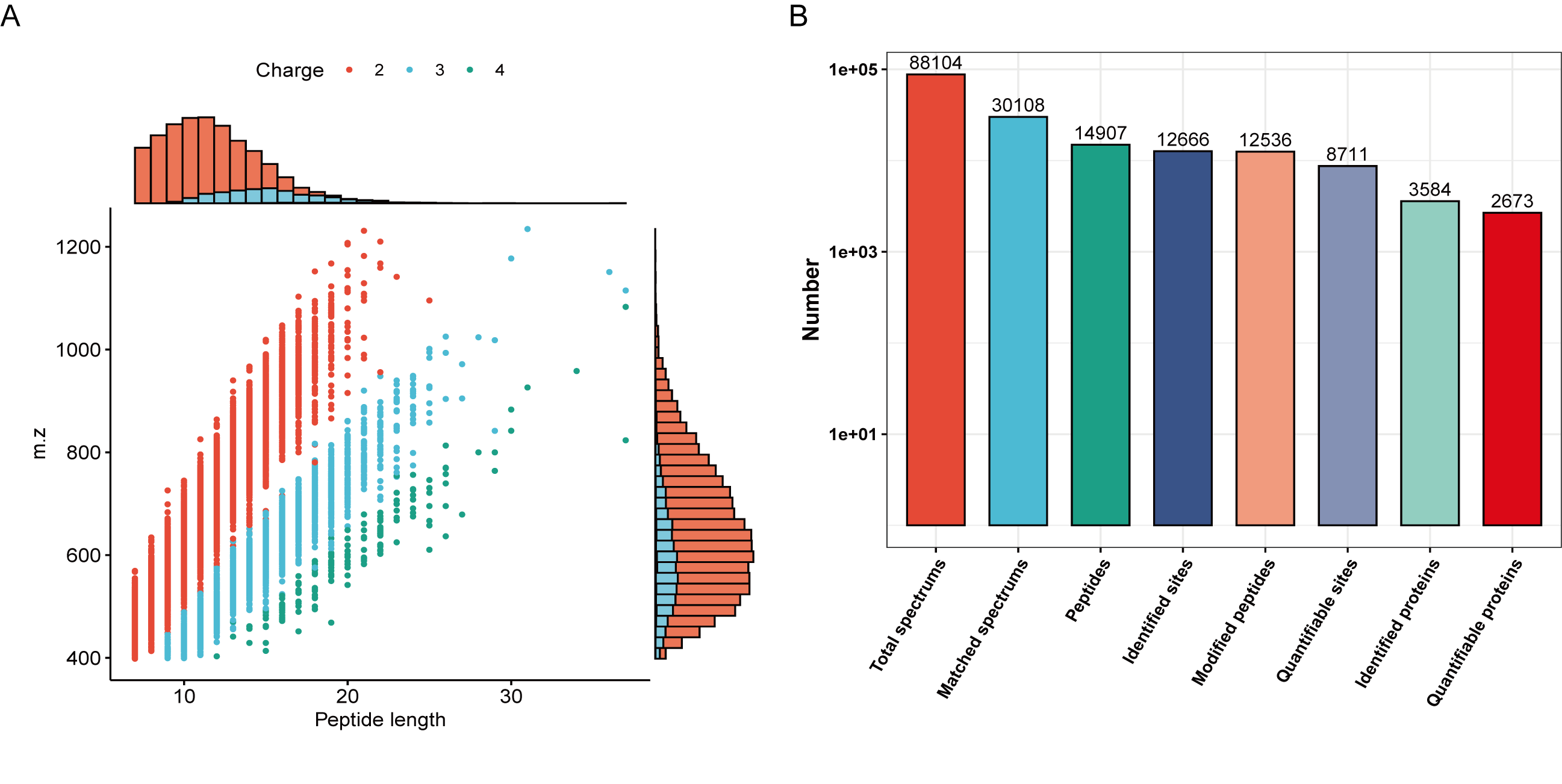

Supplement: Supplementary Figure 3 — The feature and number of peptides from crotonylation analysis. (A) The peptide length digested by trypsin. (B) The number of identified proteins. [file Image_3.tif]

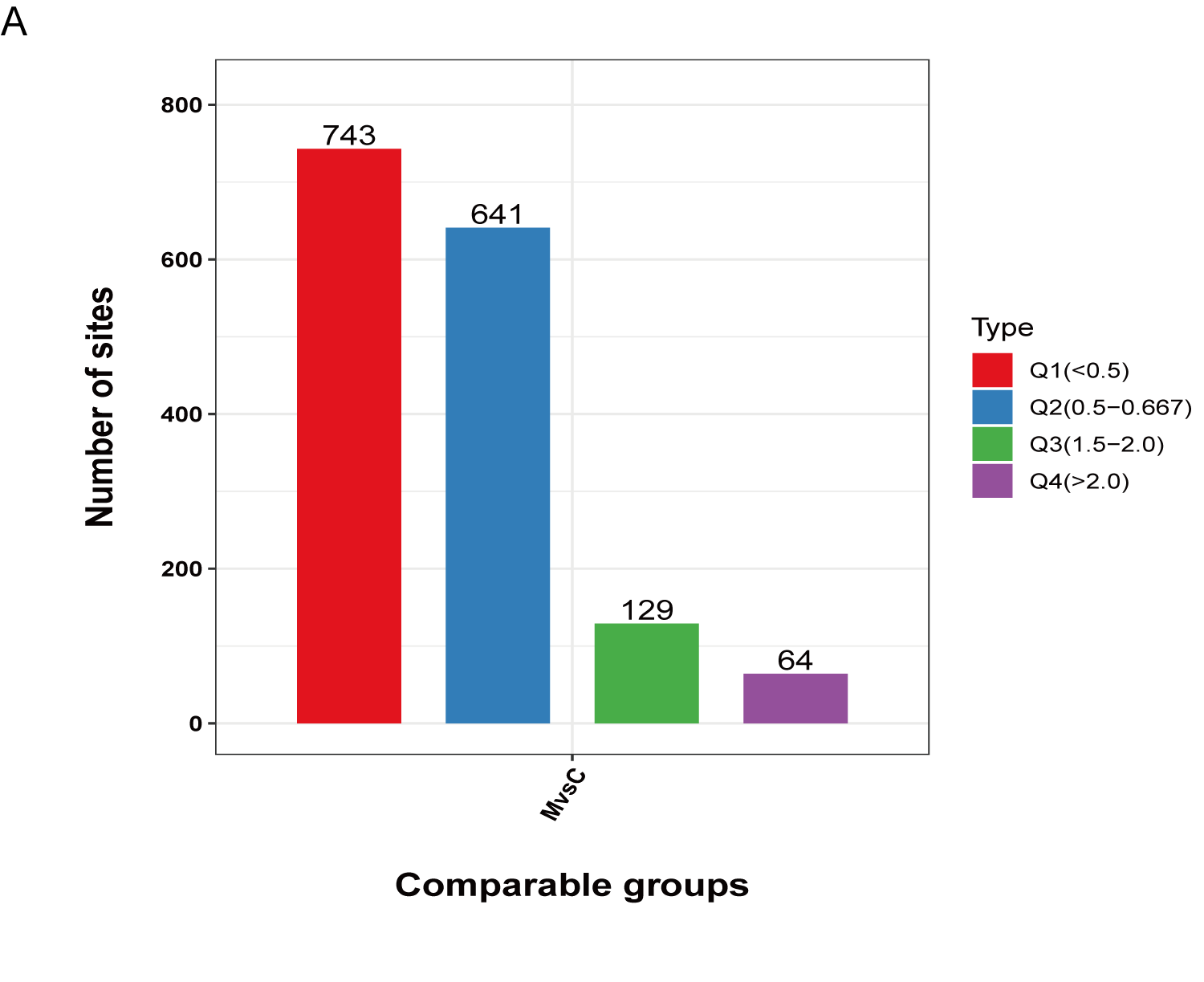

Supplement: Supplementary Figure 4 — Number of crotonylated sites from regulated peptides. [file Image_4.tif]
